# A Flexible PI/Si/SiO_2_ Piezoresistive Microcantilever for Trace-Level Detection of Aflatoxin B1 †

**DOI:** 10.3390/s21041118

**Published:** 2021-02-05

**Authors:** Yuan Tian, Yi Liu, Yang Wang, Jia Xu, Xiaomei Yu

**Affiliations:** National Key Laboratory of Science and Technology on Micro/Nano Fabrication, Institute of Microelectronics, Peking University, Beijing 100871, China; nisioisin@pku.edu.cn (Y.T.); yiliu1@pku.edu.cn (Y.L.); sunlightking@pku.edu.cn (Y.W.); xujiaa@pku.edu.cn (J.X.)

**Keywords:** polyimide, piezoresistive microcantilever, spring constant, deflection sensitivity, aflatoxin B1

## Abstract

In this paper, a polyimide (PI)/Si/SiO_2_-based piezoresistive microcantilever biosensor was developed to achieve a trace level detection for aflatoxin B1. To take advantage of both the high piezoresistance coefficient of single-crystal silicon and the small spring constant of PI, the flexible piezoresistive microcantilever was designed using the buried oxide (BOX) layer of a silicon-on-insulator (SOI) wafer as a bottom passivation layer, the topmost single-crystal silicon layer as a piezoresistor layer, and a thin PI film as a top passivation layer. To obtain higher sensitivity and output voltage stability, four identical piezoresistors, two of which were located in the substrate and two integrated in the microcantilevers, were composed of a quarter-bridge configuration wheatstone bridge. The fabricated PI/Si/SiO_2_ microcantilever showed good mechanical properties with a spring constant of 21.31 nN/μm and a deflection sensitivity of 3.54 × 10^−7^ nm^−1^. The microcantilever biosensor also showed a stable voltage output in the Phosphate Buffered Saline (PBS) buffer with a fluctuation less than 1 μV @ 3 V. By functionalizing anti-aflatoxin B1 on the sensing piezoresistive microcantilever with a biotin avidin system (BAS), a linear aflatoxin B1 detection concentration resulting from 1 ng/mL to 100 ng/mL was obtained, and the toxic molecule detection also showed good specificity. The experimental results indicate that the PI/Si/SiO_2_ flexible piezoresistive microcantilever biosensor has excellent abilities in trace-level and specific detections of aflatoxin B1 and other biomolecules.

## 1. Introduction

Aflatoxin B1 is a type of aflatoxin produced by fungi such as Aspergillus flavus and Aspergillus parasiticus, and is the most toxic substance of aflatoxin. The median toxic dose (TD50), which is highly carcinogenic and is found in half of the laboratory animals, is 0.0032 mg/kg/day in rats [1]. Aflatoxin B1 can be a pollutant in various foods such as lacquer, corn, cottonseed meal, grains, and animal feed. Tolerable levels of aflatoxin B1 have been set by the Food and Agriculture Organization of the United Nations (FAO), with concentrations of 1 to 20 µg/kg in food and 5 to 50 µg/kg in edible cattle feed [2,3,4,5]. Hence, real-time, label-free, and trace-level detections for aflatoxin B1 represent an important and serious issue to ensure food safety.

In recent years, several traditional analytical techniques, such as high-performance liquid chromatography–mass spectrometry (HPLC-MS) [6,7], have been introduced for aflatoxin B1 detection. These techniques offer a high sensitivity and stability detection of aflatoxin B1. However, these analytical techniques often require expensive and bulky instruments and sophisticated laboratory environment. Thus, several biosensors have been developed, such as immunoassay [8,9,10,11], surface plasmon resonance sensor [12], electrochemical sensor [13,14] and surface-enhanced Raman spectroscopic (SERS) sensor [15], fluorescence sensor [16,17], chemiluminescence (CL) sensor [18,19,20,21], imprinted polymer membrane sensor [22], and microcantilever sensor [23]. Compared to other sensor techniques, microcantilever is an ultrasensitive and rapid MEMS device for biochemical sensing. In 2016, Zhou et al. reported a nanomechanical high-sensitivity aflatoxin B1 detection, and a 1 ng/mL aflatoxin B1 detection in a peanut solution was achieved [23]. Although a high detection limit was obtained, the readout method was optical, which also needs a bulky and high-cost optical system, and often requires technical expertise to operate. As an alternative to the static cantilever deflection, carrying out a dynamic excitation of the cantilever and tracking its resonance frequency has been proposed for liquid-phase bio/chemical detection in the literature. In this case, the cantilever can be operated with a large Q-factor in air, and solute detection is possible with a resonance frequency shift [24], using a microfluidic platform requiring only 30 µL of solution [25], drop-casting of drops with a volume of only 49.7 ± 1.9 pL on a cantilever [26], or imprinting a solute on a cantilever [27]. 

A resistance or voltage signal of a piezoresistive microcantilver can easily be read with a high-precision multimeter or an analog–digital converter. As it can convert a biochemical signal to an electrical signal, the piezoresistive microcantilever biosensor is a simple and valid way to conduct biochemical detections. The piezoresistive microcantilever usually uses single-crystalline silicon as a piezoresistive material, and silicon dioxide or silicon nitride as its passivation layers. In 2015, Zhao et al. reported a high SNR piezoresistive microcantilever biosensor to detect abrin with a sensitivity of 0.08 ng/mL in orange juice and skim milk by using single-crystal silicon as a piezoresistor [28]. In 2019, Yen et al. reported a silicon-based microcantilever for gentamicin detection with a minimum detection concentration of 9.44 μg/mL [29]. 

The spring constant of the microcantilever is closely related to the type of material being studied. The smaller the spring constant, the more deflection-sensitive the microcantilever. Compared to semiconductor dielectric materials, polymers have a lower spring constant, while most of them can be processed with MEMS techniques; therefore, polymer-based microcantilever have received attention from researchers in recent years. In 2017, Kim et al. demonstrated a polydimethylsiloxane (PDMS) piezoresistive microcantilever biosensor. The microcantilever biosensor measured the cardiomyocytes to detect Verapamil with a concentration from 50 nM to 1 μM [30]. In 2018, Sun et al. studied a parylene-based microcantilever with poly (3,4-ethylenedioxythiophene)/poly (styrene sulfonate) (PEDOT/PSS) as a piezoresistor. The spring constant and the deflection sensitivity were 17 nN/μm and 8.59 × 10^−7^ nm^−1^, respectively. The IgG detection limit was 10 ng/mL [31]. In 2020, our group proposed a low spring-constant polyimide(PI)-based microcantilever biosensor, which consisted of a four-fold titanium piezoresistor and a PI passivation layer [32]. For titanium, the resistance change is mainly caused by dimension change; thus, the deflection sensitivity is usually lower than singlecrystal silicon as the piezoresistive material. Compared to the conference paper [33], a new microcantilever biosensor was fabricated with different wheatstone bridge configurations and photolithography masks to improve both mechanical and electrical performance. This paper expands on biosensor design, fabrication process, biochemical molecules detection, and specific detection.

To ensure a high sensitivity and low spring-constant of the piezoresistive microcantilever, we propose a piezoresistive microcantilever in which the single-crystal silicon layer of a silicon-on-insulator (SOI) wafer was used as the piezoresistor and a thin PI layer as the top passivation layer in this work. With the fabricated microcantilever biosensor, aflatoxin B1 with a minimum concentration of 1 ng/mL was detected by using the biotin avidin system (BAS) method.

## 2. Design and Fabrication

### 2.1. Design of the PI/Si/SiO_2_ Microcantilever

Surface stresses occur during the coupling process between the probe and the target, resulting in bending of the microcantilever and changes in resistance. The relative change in piezoresistive resistance (ΔR/R) can be expressed as follows:

(1) $$\;\Delta R/R\;=\;\left(1\;+\;2v\right)\varepsilon \;+\;\Delta \rho /\rho_{0}$$

where v is Poisson’s ratio of the piezoresistive material; ε is the relative change in length of the piezoresistor; and ∆ρ and ρ_0_ are the electrical resistivity change and the original electrical resistivity of the piezoresistor, respectively. For a single-crystal silicon piezoresistor, $\Delta \rho /\rho_{0}$ is much higher than the dimension effect under the same stress, and plays a major role in ΔR/R [34].

To take advantage of the high piezoresistive coefficient of single-crystal silicon, the buried oxide (BOX) layer SiO_2_ and the single-crystal silicon layer of a SOI wafer were used as the bottom passivation layer and piezoresistor, respectively. PI is a flexible polymer material commonly used in MEMS technology. It is resistant to acids, alkalis and organic solvents after curing into a film, and has good insulation and other advantages. In this work, PI 2610 with low stress, a low thermal-expansion coefficient, low water absorption, and a Young’s modulus of 8.5 GPa was selected as the top passivation layer of the microcantilever. To ensure both a high sensitivity and a low spring constant, which ensure the microcantilever is not easily broken, a 1.2 μm thick PI layer was used as the top passivation layer to protect the piezoresistor. The PI/Si/SiO_2_ microcantilever is designed as rectangle with a dimension of 120 μm × 50 μm, while the embedded piezoresistor dimension is 60 μm × 15 μm in a two-fold structure.

**Figure 1:**
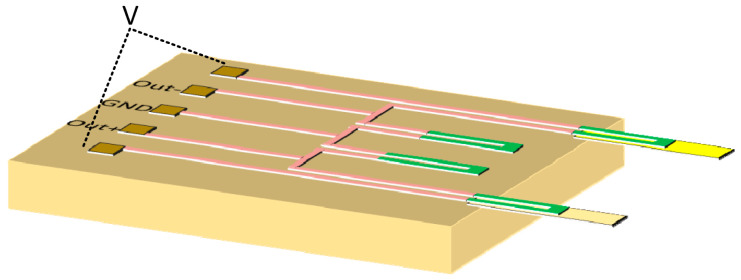
The schematic diagram of the quarter-type wheatstone bridge.

As shown in Figure 1, four identical single-crystal silicon piezoresistors, two of which were located in the substrate and two integrated in the microcantilevers, constituted a Wheatstone bridge. A Cr/Au-modified layer was coated on the top PI layer of one microcantilever, which served as a sensing microcantilever for specific biotinylated polyclonal antibody (bio-PcAb) immobilization. The second microcantilever served as a reference cantilever. When an input voltage V_in_ was applied to the Wheatstone bridge, a voltage output V_out_ of the Wheatstone bridge occurred as follows:

(2) $${\;V}_{\mathrm{out}}\;=\;0{.25\;\times \;V}_{\mathrm{in}}\times \Delta R/R$$

### 2.2. Fabrication Process

**Figure 2:**
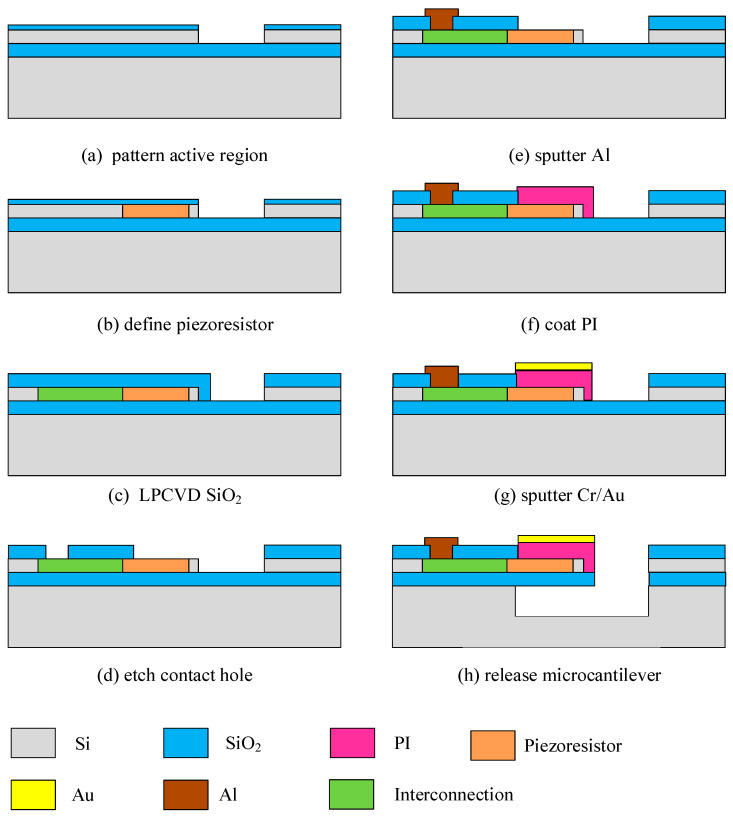
The fabrication processes of PI/Si/SiO_2_ microcantilevers.

The microcantilever shown above was fabricated using four-inch SOI wafers. The main parameters of the SOI substrate were as follows: the BOX layer thickness was 400 nm, the single-crystal silicon layer was P-type in [100] orientation, and the thickness was 340 nm. Polyimide (PI 2610, Microsystem) with a Young’s modulus of 8.5 GPa was used as the top passivation layer. After finishing all planar processes, the microcantilever was released by a hybrid process of anisotropic and isotropic dry etching to avoid microcantilever damage or contamination. The fabrication process is shown in Figure 2 and depicted as follows:

(a) A thermal oxidation method was used to form a layer of 30 nm-thick SiO_2_ on the surface of the SOI wafer for the ion-implantation protective layer, and then the single-crystal layer was inverted to form an N^+^ ring using phosphorus-ion implantation. The implant energy was 100 keV and the implant dose was 5 × 10^13^ cm^−2^. Then, the first photolithography was performed, and the patterned single-crystal silicon layer was dry-etched by reactive ion etching (RIE) to form an active region.
(b) The two-fold piezoresistors were patterned by the second photolithography and defined by boron-ion implantation. The implant energy was 100 keV and the implant dose was 4 × 10^14^ cm^−2^. The resistance of the piezoresistor was approximately 5 kΩ.
(c) High-concentration boron-ion implantation formed a heavily doped area as the interconnection of the piezoresistors and the contact pads by the third photolithography. The implant energy was 100 keV and the implant dose was 8 × 10^15^ cm^−2^. The implantation ions were activated under annealing conditions of 1050 °C and 30 s. A 200 nm SiO_2_ was deposited as a passivation dielectric layer of the active region by a low pressure chemical vapor deposition (LPCVD).
(d) The metal contact hole was opened by buffer hydrofluoric acid (BHF) wet-etching by the fourth photolithography.
(e) An 800 nm aluminium contact pad was sputtered on the surface of the SOI wafer, and patterned by the fifth photolithography. Then, the SOI wafer was alloyed at 470 °C in a nitrogen environment to form ohmic contact pads.
(f) A 1.2 μm polyimide film was spun on the surface of the SOI wafer, which was only kept on the top of the microcantilever by oxygen-plasma etching after the sixth photolithography.
(g) A 10/50 nm Cr/Au layer was sputtered on the surface of the SOI wafer, and lifted off to form the modified layer of functionalization on the surface of the sensing microcantilever by the seventh photolithography.
(h) After the eighth photolithography with a 3 μm thick photoresist as a mask, the SiO_2_ inside the reactive well area was totally etched away until the silicon substrate was completely exposed. After an anisotropic dry-etching was introduced to etch the silicon substrate for 10 μm, an isotropic dry-etching was used to continue etching the silicon substrate until the microcantilever structure was completely released.

Several improved processes were introduced to reduce the output signal fluctuations during the fabrication. Firstly, a N^+^-type ring was introduced around the piezoresistor to reduce current leakage of the piezoresistor. Secondly, the electrical interconnection between the contact pads and the piezoresistor was defined by a high concentration boron-ion implantation on the Si-device layer rather than the metal interconnections, thereby effectively reducing the thermal drift of the sensor. Thirdly, instead of a potassium hydroxide (KOH) wet-etching, a mixed process of isotropic and anisotropic dry silicon etching was used to avoid potassium-ion contamination and microcantilever beam fracture.

**Figure 3:**
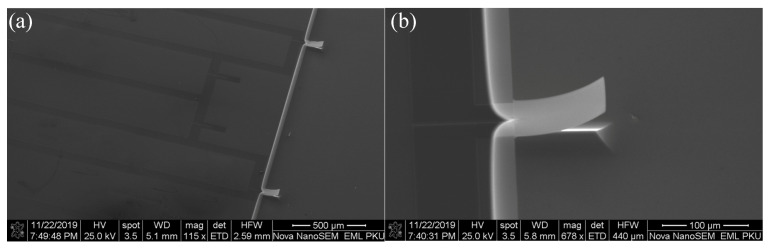
(**a**,**b**) are SEM photos of a microcantilever array and a single microcantilever, respectively.

About 400 microcantilever biosensors were fabricated on five four-inch SOI wafers in one manufacturing process. A scanning electronic microscopy (SEM) photograph of two piezoresistors located on the substrate and two piezoresistors integrated on the released microcantilevers constituting a wheatstone bridge is shown in Figure 3a, and Figure 3b is an SEM photograph of a single sensing microcantilever. It can be seen from Figure 3 that there is an intrinsic bending deformation for the microcantilever since it was designed in multiple layers. Due to factors such as the mismatch effect of the thermal expansion coefficient between the various layers of the film, the residual stress generated inside the microcantilever causes it to undergo intrinsic deformation and bending.

**Figure 4:**
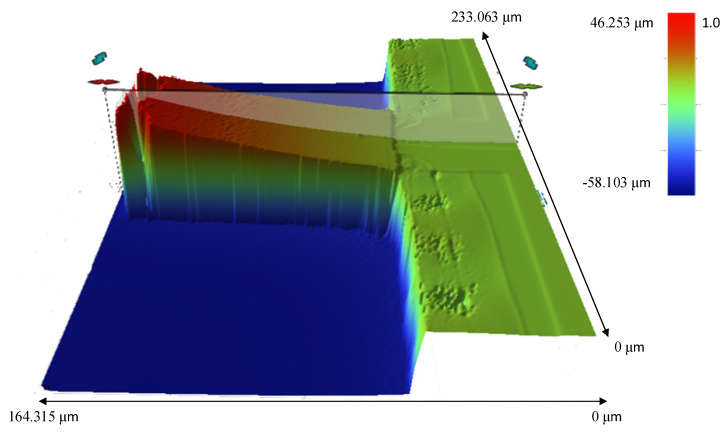
The 3D measurement image of a fabricated microcantilever by a 3D optical microscopy. The upper bend height of the free end of the microcantilever is 46.253 μm and the depth of the reactive well is 58.103 μm.

To obtain the precise bending height of the microcantilever, a 3D image of the microcantilever was obtained by an optical profiler. As shown in Figure 4, the microcantilever bends 46.253 μm upward, and the depth of the reactive well is 58.103 μm.

## 3. Properties of the Microcantilever

### 3.1. Spring Constant

The spring constant k of a single layer rectangular microcantilver can be calculated as [35]:

(3) $$k\;=\;\frac{{\mathrm{Ewt}}^{3}}{{4l}^{3}}$$

where E is the Young’s modulus of the material; w, t, and l are the width, thickness, and length of the microcantilver, respectively. The spring constant for a multi-layer microcantilever processed in different experimental environments is difficult to be calculated due to the complex matching and non-ideal Young’s modulus of different layers. So, it is necessary to calibrate the actual spring constant of the composite layer microcantilever experimentally before a biological molecule detection can occur.

The spring constant of the PI/Si/SiO_2_ microcantilever was calibrated by a reference atomic force microscopy (AFM) microcantilever. The accurate spring constant value of microcantilever can then be calculated by the relative displacement of the AFM tip and the microcantilever deflection.

(4) $$k\;=\;\frac{\Delta z}{\Delta h}k_{\mathrm{tip}}$$

where k is the spring constant of the microcantilever, Δz is the relative displacement of the AFM tip, Δh is the relative displacement of the microcantilever, and k_tip_ is the spring constant of the AFM tip.

**Figure 5:**
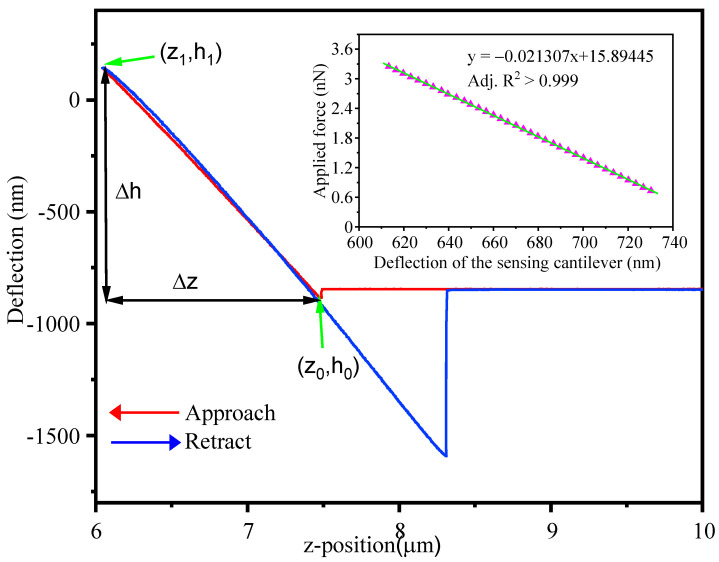
The correlation between the microcantilever deflection and the AFM tip displacement, The red approach curve is used for calculation. Inset is the relationship between the applied force and the deflection of the sensing microcantilever.

In this work, an AFM tip with a spring constant of 80 nN/μm and a resonance frequency of 34 kHz was used during the spring constant measurement. Figure 5 shows a linear correlation between the microcantilever deflection and the AFM tip displacement. A spring constant of 21.307 nN/μm was calibrated in the approach curve from (z_0_, h_0_) point to (z_1_, h_1_) point for the microcantilever which is approximately half of the spring constant of 46.79 nN/μm for the SiO_2_/Si/SiO_2_ microcantilevers [5].

### 3.2. Sensitivity

**Figure 6:**
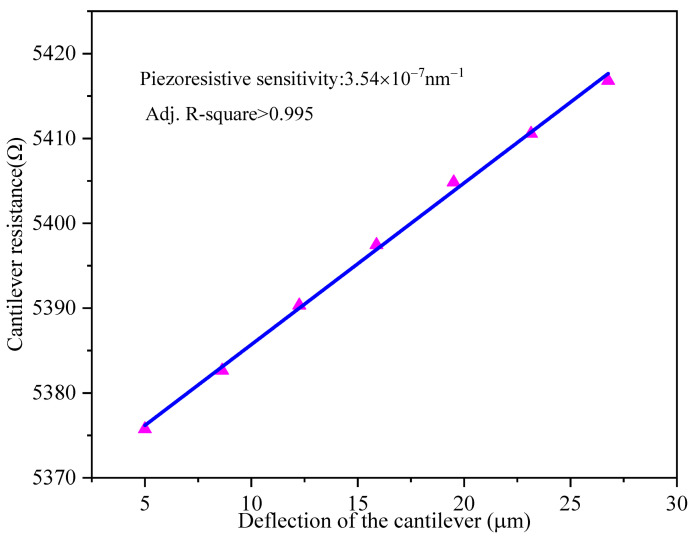
The sensing microcantilever resistance change vs. deflection of the free end for PI/Si/SiO_2_ microcantilever.

The deflection sensitivity of the microcantilever is defined as ΔR/R∙Δz^−1^. In order to test the deflection sensitivity of the microcantilever, a metal probe was fixed on a precision electronically controlled translation platform, which is controlled by LabView. During the experimental process, the microcantilever sensor chip was fixed on the platform through a fixture, and the metal probe, which put the free end of the microcantilever downward, was fixed on the platform. The resistance change of the piezoresistor was measured using an Agilent 34401A 61⁄2 high-precision multimeter. Figure 6 shows the resistance change vs. deflection change of the free end Δz of the PI/Si/SiO_2_ microcantilever. The deflection sensitivity of the microcantilever was then set at 3.54×10^−7^ nm^−1^ from the slope of the relationship curve and the correlation was linear with an adjusted R-squared of 0.99, which indicates that the PI/Si/SiO_2_ microcantilever can meet the requirements of the trace-level detection of biomolecules such as aflatoxin B1.

The value of the piezoresistive gauge factor can be calculated by Equation (5) of reference [36]:

(5) $$K\;=\;\frac{\Delta R}{R}/\Delta z\times \frac{{2l}_{c}^{3}}{{3t(l}_{c}-0{.5l}_{p})}\;$$

where K is the gauge factor, ΔR/R∙Δz^−1^ is the deflection sensitivity; t is the thickness of the microcantilever; l_c_ and l_p_ are the length of the microcantilever and the piezoresistor, respectively. The gauge factor is then calculated as 23.357.

### 3.3. Output Stability in PBS Buffer

**Figure 7:**
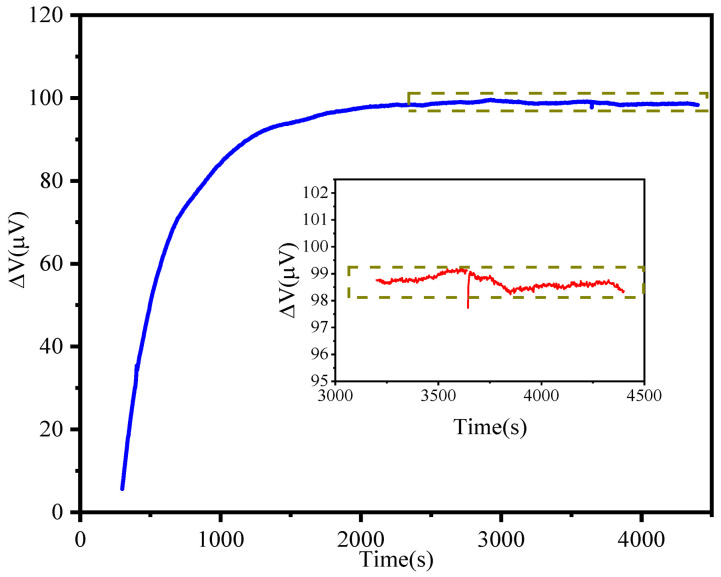
The stability measurement result of the microcantilever in PBS buffer with 3 V bias voltage. Inset is a y-coordinated enlarged figure, the output voltage fluctuation is between 98 and 99 μV.

In order to intuitively reflect the minimum detection limit that the microcantilever biosensor can achieve in a liquid environment, the stability of the microcantilever output signal in a Phosphate Buffered Saline (PBS) buffer environment was tested. The test system consisted of an Agilent 34401A 61/2 high-precision multimeter, a 3 V power supply, and a liquid reaction well. During the experiment, the microcantilever chip was placed in a 0.01 M PBS at room temperature. After applying the 3 V bias voltage, the output signal curve was obtained by the Agilent 34401A 61/2 high-precision multimeter. The output voltage fluctuation can be obtained when the output signal reaches a stable level. Figure 7 shows the test result of the output signal fluctuation of the microcantilever in the PBS buffer at room temperature. It can be seen from the figure that the output signal rised rapidly within the first 1000 s from the beginning of the experiment. This is caused by the self-heating effect of the sensitive resistor and the different thermal expansion coefficients of the composite material of the microcantilever. Based on the view of J. C. Doll et al. [37], the magnitude of the self-heating effect depends on the sensor geometry, the thermal conductivity of the materials used, and the thermal properties of any surrounding fluid. The thermal conductivity of water is approximately 25 times greater than that of air. Therefore, the microcantilever requires a quite long time to reach the heat equilibrium. After 1500 s, the output signal gradually reached a stable state. Now, the temperature cross sensitivity played a minor role, while the 1/f noise and Johnson noise of the piezoresistors played a leading role. At the same time, environmental mechanical noise and power supply voltage fluctuations also contributed a large part of the fluctuation. From the inset of a y-coordinated enlarged figure in Figure 7, it can be seen that the output signal fluctuation was basically maintained in the range of 1 μV at a bias voltage of 3 V, which shows that the PI/Si/SiO_2_ piezoresistive microcantilever has extremely stability that can fully meet the high-precision trace-level detection of biochemical molecules.

## 4. Experiment Preparation

### 4.1. Reagents and Materials

The PBS solution for the experiment was purchased from M&C Gene Technology Ltd. (Beijing, China). The 3,3′-Dithiopropionic acid (DDPA), N-Hydroxy Succinimide (NHS), 1-ethyl-3-(3-dimethylaminopropyl)-carbodiimide hydrochloride (EDC), ethanolamine, streptavidin, biotinylated goat anti-human IgG, and human IgG were purchased from Sigma-Aldrich Co. LLC (Beijing, China). Aflatoxin B1 was purchased from Acros Organics (Beijing, China). Biotinylated aflatoxin B1 antibody was purchased from Thermo Fisher Scientific (Shanghai, China). Ricin and abrin for specific testing were received from Dianotech Science and Technology Co., Ltd. (Beijing, China). Ethanol, acetone, and deionized water were all provided by the National Key Laboratory of Science and Technology on Micro/Nano Fabrication, Microelectronics Technology Laboratory of Peking university.

### 4.2. Surface Functionalization

A biotin avidin system (BAS) was introduced into the surface functionalization of the microcantilever for aflatoxin B1 detection. Biotin is composed of two parts—a thiophene ring with a carboxyl group, and an imidazole ring. The thiophene ring can effectively bind different antibodies and aptamer probes to realize the biotinylation of the probe; the imidazole ring is the main component that binds to avidin. Generally, after the probe molecule is biotinylated, its binding activity to the target molecule is not affected. Therefore, biotin is widely used in biochemical sensing and detection technology. At present, the commonly used avidin in the BAS method is streptavidin extracted from Streptomyces. Avidin is a protein composed of four subunits with the same sequence. Each subunit can be combined with a biotin molecule. After biotin and avidin are combined, a complex structure similar to a lattice is formed. The binding specificity between the two is extremely strong, and the affinity (the association constant is as high as 10^15^ mol/L) is more than ten thousand times that of the antigen-antibody (the association constant between 10^5^ and 10^11^ mol/L). Due to the strong binding between biotin and avidin and the multi-stage amplification effect, the biochemical detection using BAS can achieve a higher sensitivity.

The basic steps of the surface functionalization are as follows: (1) Immerse the pre-treated microcantilever biosensor in 5 mg/mL DDPA for about 1 h to coat the Au film on the surface of the sensing microcantilever with carboxyl groups; (2) Take out the microcantilever biosensor from DDPA and wash it several times, and then immerse it in a mixture of EDC/NHS with a concentration of 5 mg/mL and a volume ratio of 3:1 for about 30 min to form succinimide activators on the surface of the sensing microcantilever; (3) After repeatedly cleaning the microcantilever biosensor, immerse it in a 0.1 mg/mL avidin solution and react for about 1 h to cross-link the avidin molecule with the succinimide activator; (4) Clean the microcantilever biosensor several times, then add 1 M ethanolamine to inactivate the residual carboxyl groups on the surface of the sensitive beam—the reaction time is 30 min; (5) After cleaning the microcantilever biosensor several times, immerse it in the PBS solution until the sensor output is stable. Then add different concentrations and different types of biological probes until the microcantilever biosensor output voltage signal is stable. All of the proposed procedures were operated at room temperature.

## 5. Results and Discussions

### 5.1. IgG Detection

For the IgG detection, the PI/Si/SiO_2_ flexible piezoresistive microcantilever functionally modified by the BAS method was immersed in the biotinylated goat anti-human IgG with a concentration of 2 mg/mL at room temperature. The output signal is related to the number of goat anti-human IgG antibodies bound to the surface of the sensor chip. In order to make the entire area of the gold surface bind to the biotinylated goat anti-human IgG as much as possible and to achieve a lower detection concentration limit, it is necessary to ensure the antibody concentration enough for surface functionalization. The microcantilever chip is incubated in goat anti-human IgG for 1 h and then washed away the goat anti-human IgG that has not been modified on the microcantilever gold surface, and finally the construction of an immunosensor for detecting IgG is completed.

**Figure 8:**
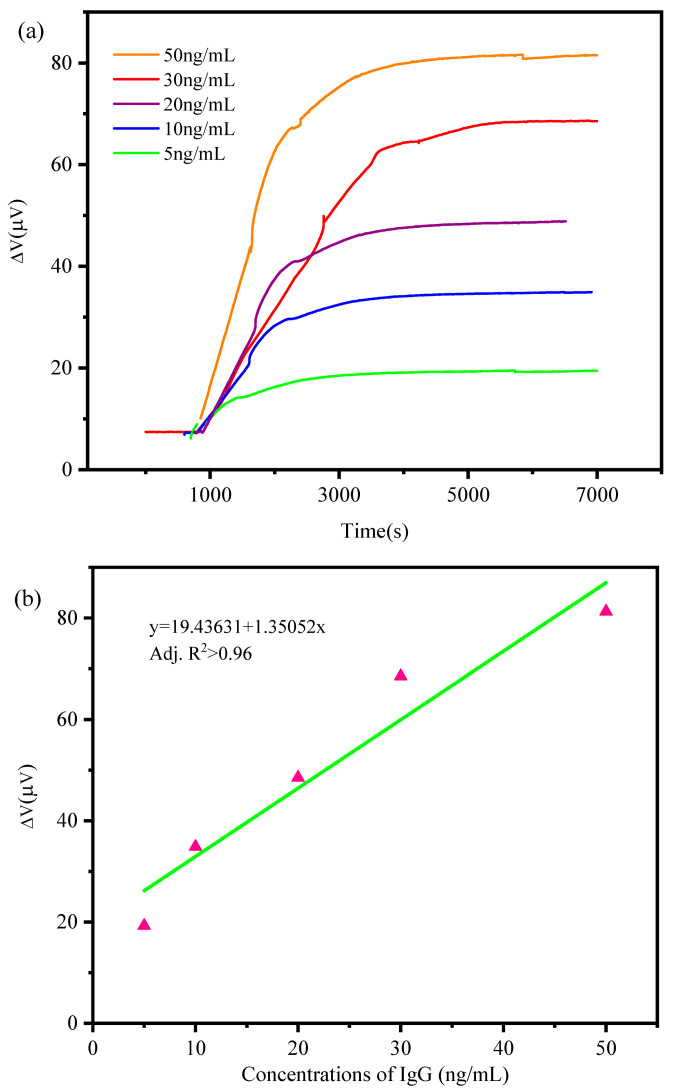
(**a**) The measurement results of IgG at concentrations of 5, 10, 20, 30, 50 ng/mL, respectively. (**b**) a linear correlation of the measured output voltage response and IgG concentrations. Fitting function is y = 19.43631 + 1.36052x. Adj. R^2^ > 0.96 for fitting curves.

During the experiment, the microcantilever chip was immersed in a reaction well with a volume of 550 μL vertically. After the output signal was stable, different concentrations of human IgG were added to the reaction well. The final detection concentration of human IgG in the detection well was 5 ng/mL, 10 ng/mL, 20 ng/mL, 30 ng/mL, 50 ng/mL. Figure 8a shows the curve of the output voltage signal of the microcantilever vs. time caused by human IgG detection at different concentrations. After adding different concentrations of human IgG, the sensing microcantilever beam began to deform, and the resistance value of the piezoresistor changed, so the output voltage of the formed Wheatstone bridge began to increase. The higher the concentration of human IgG, the greater the output voltage of the microcantilever, and the saturation time eventually reached was also longer. As shown in Figure 8b, the output voltage of the microcantilever chip showed a good linear relationship with the concentrations of human IgG. Based on the 1 μV output voltage fluctuation, a limit of detection (LOD) was calculated to be 0.19 ng/mL from the fitting curves slope in Figure 8b.

### 5.2. Aflatoxin B1 Detection

Approximately thirty microcantilevers were surface functionalized for the detection of aflatoxin B1 for one batch of experiments. The PI/Si/SiO_2_ flexible piezoresistive microcantilever was also functionalized by the BAS method. After streptavidin was connected to the surface of the sensing microcantilever, the PI/Si/SiO_2_ flexible piezoresistive microcantilever was immersed in the PBS buffer with biotinylated aflatoxin B1 antibody at a concentration of 30 μg/mL; after incubating for one hour at room temperature, the surface of the sensing cantilever was connected with biotinylated aflatoxin B1 antibody. Finally, the surface modification was completed to form a biosensor capable of detecting aflatoxin B1.

**Figure 9:**
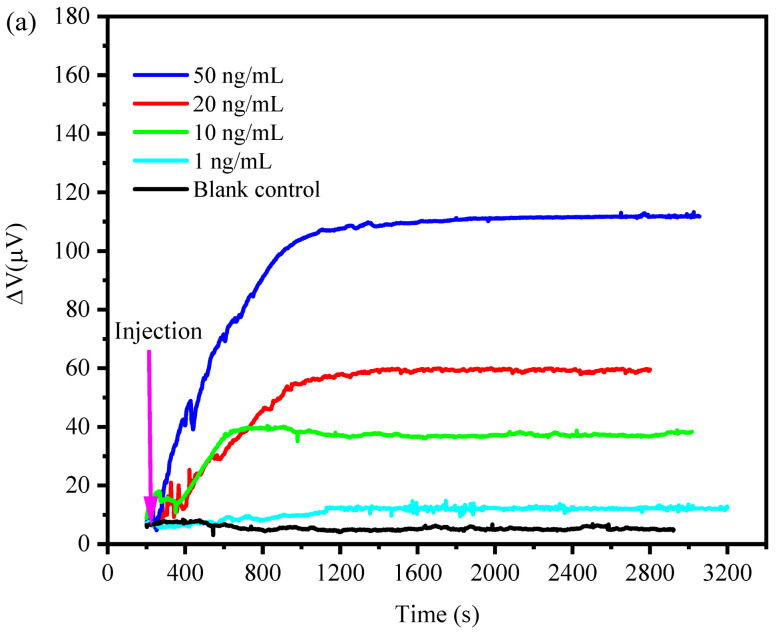

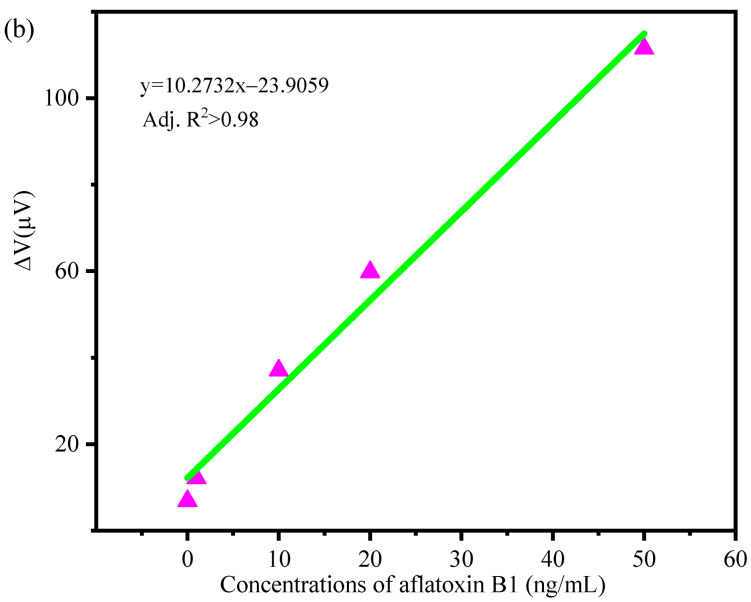
(**a**) the measurement results of aflatoxin B1 at concentrations of 1, 10, 20, 50 ng/mL, respectively. (**b**) a linear correlation of the measured output voltage response and aflatoxin B1 concentrations. Fitting function is y = 10.2732x − 23.9059. R^2^ > 0.98 for fitting curves.

For the Aflatoxin B1 detection, the modified PI/Si/SiO_2_ flexible piezoresistive microcantilever biosensor was placed vertically into the reaction well of 550 μL with a concentration of 0.01M PBS buffer at room temperature. When the output signal was stable, different concentrations of aflatoxin B1 solution were injected into the detection well with the diluted concentrations of 1 ng/mL, 10 ng/mL, 20 ng/mL, and 50 ng/mL, respectively. Figure 9a shows the relationship between the output signal and time of aflatoxin B1 detection by the PI/Si/SiO_2_ flexible piezoresistive microcantilever sensor. It can be seen that the PI/Si/SiO_2_ flexible piezoresistive microcantilever sensor can complete the detections within 1000 s at the four given detection concentrations. Obviously, the higher the aflatoxin B1 concentration, the higher the saturation value of the output voltage and saturation time. This is because, under higher concentration detection, more aflatoxin B1 molecules can be absorbed and bound to the aflatoxin B1 antibody, causing greater surface stress. At the same time, because more aflatoxin B1 molecules bound to the antibodies on the surface of the sensing microcantilever, it takes more time for the biosensor to reach the dynamic balance of the output voltage.

Figure 9b shows the correlation between the measured output voltage response and aflatoxin B1 concentrations. By fitting the relationship curve between the output voltage of the PI/Si/SiO_2_ flexible piezoresistive microcantilever and the concentration of aflatoxin B1, an Adj.R^2^ of 0.98 was obtained, and the lowest detection concentration reached 1 ng/mL. Based on the 1 μV output voltage fluctuation, the LOD of Aflatoxin B1 was calculated as 0.1 ng/mL. Compared to optical readout microcantilever biosensor [23], the same level of LOD was obtained.

Three measurements were usually carried out by different microcantilever biosensors for each concentration of the aflatoxin B1 detection since the bounded aflatoxin B1 was difficult to be removed from the surface of microcantilever biosensor. Since the microcantilevers were functionalized at the same time and under the same conditions, the results of the experiments can be repeated well with the same concentration detection.

### 5.3. Specific Detections

**Figure 10:**
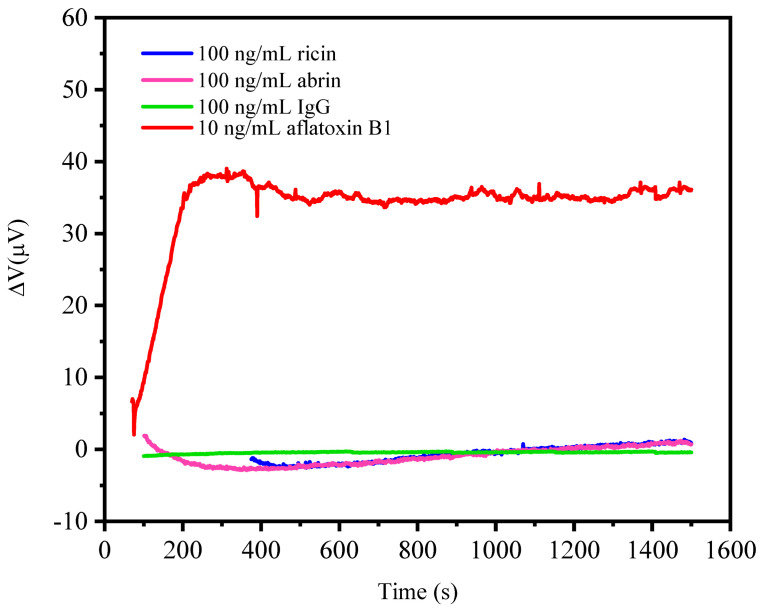
The specific detection results of ricin, abrin, and IgG at concentrations of 100 ng/mL by microcantilever biosensors functionalized with aflatoxin B1 antibody.

All of the biomolecule detections are expected to have a good specificity. So, specific detection experiments were proceeded with three control groups to observe the output voltage variation. Before the specificity detections, the microcantilevers were modified with aflatoxin B1 antibody under the same condition and the same concentration. After that, the microcantilever biosensors were put into the PBS buffer until the output voltage signal reached a dynamic balance. Then, ricin, abrin, and IgG with a concentration of 100 ng/mL were added to the reactive well separately to test the specificity of the microcantilever biosensor. As shown in Figure 10, the control group output voltage variation is almost zero, even when the control concentrations are as high as 100 ng/mL, while the output voltage changes to 38 μV for detecting aflatoxin B1 at a concentration of 10 ng/mL due to the binding process of aflatoxin B1 and its antibody. These results prove that our microcantilever biosensor has an excellent specificity.

## 6. Conclusions

In this paper, we developed a PI/Si/SiO_2_-flexible piezoresistive microcantilever for the trace-level detection of biochemical molecules such as IgG and aflatoxin B1 with excellent specificity. The fabricated PI/Si/SiO_2_ microcantilever showed good mechanical properties with a spring constant of 21.31 nN/μm and a deflection sensitivity of 3.54 × 10^−7^ nm^−1^. The microcantilever biosensor also showed a stable voltage output in the PBS buffer with a fluctuation less than 1 μV @ 3 V for the Wheatstone bridge. The IgG detection showed a minimum detection concentration of 5 ng/mL with a good linear correlation of 0.96 adjusted R-squared. The aflatoxin B1 detection showed a minimum detection concentration of 1 ng/mL with a good linear correlation of 0.98 adjusted R-squared, while the LOD reached 0.1 ng/mL. The PI-based piezoresistive biosensor showed a good flexibility compared with SiO_2_/Si/SiO_2_ during the experiments, which will improve the detection reliability at different application environments.
